# Machine Learning Models for Diagnosis of Parkinson’s Disease Using Multiple Structural Magnetic Resonance Imaging Features

**DOI:** 10.3389/fnagi.2022.808520

**Published:** 2022-04-13

**Authors:** Yang Ya, Lirong Ji, Yujing Jia, Nan Zou, Zhen Jiang, Hongkun Yin, Chengjie Mao, Weifeng Luo, Erlei Wang, Guohua Fan

**Affiliations:** ^1^Department of Radiology, The Second Affiliated Hospital of Soochow University, Suzhou, China; ^2^Institute of Advanced Research, Infervision Medical Technology Co., Ltd, Beijing, China; ^3^Department of Neurology, The Second Affiliated Hospital of Soochow University, Suzhou, China

**Keywords:** Parkinson’s disease, machine learning, structural magnetic resonance imaging, logistic regression, external validation

## Abstract

**Purpose:**

This study aimed to develop machine learning models for the diagnosis of Parkinson’s disease (PD) using multiple structural magnetic resonance imaging (MRI) features and validate their performance.

**Methods:**

Brain structural MRI scans of 60 patients with PD and 56 normal controls (NCs) were enrolled as development dataset and 69 patients with PD and 71 NCs from Parkinson’s Progression Markers Initiative (PPMI) dataset as independent test dataset. First, multiple structural MRI features were extracted from cerebellar, subcortical, and cortical regions of the brain. Then, the Pearson’s correlation test and least absolute shrinkage and selection operator (LASSO) regression were used to select the most discriminating features. Finally, using logistic regression (LR) classifier with the 5-fold cross-validation scheme in the development dataset, the cerebellar, subcortical, cortical, and a combined model based on all features were constructed separately. The diagnostic performance and clinical net benefit of each model were evaluated with the receiver operating characteristic (ROC) analysis and the decision curve analysis (DCA) in both datasets.

**Results:**

After feature selection, 5 cerebellar (absolute value of left lobule crus II cortical thickness (CT) and right lobule IV volume, relative value of right lobule VIIIA CT and lobule VI/VIIIA gray matter volume), 3 subcortical (asymmetry index of caudate volume, relative value of left caudate volume, and absolute value of right lateral ventricle), and 4 cortical features (local gyrification index of right anterior circular insular sulcus and anterior agranular insula complex, local fractal dimension of right middle insular area, and CT of left supplementary and cingulate eye field) were selected as the most distinguishing features. The area under the curve (AUC) values of the cerebellar, subcortical, cortical, and combined models were 0.679, 0.555, 0.767, and 0.781, respectively, for the development dataset and 0.646, 0.632, 0.690, and 0.756, respectively, for the independent test dataset, respectively. The combined model showed higher performance than the other models (Delong’s test, all p-values < 0.05). All models showed good calibration, and the DCA demonstrated that the combined model has a higher net benefit than other models.

**Conclusion:**

The combined model showed favorable diagnostic performance and clinical net benefit and had the potential to be used as a non-invasive method for the diagnosis of PD.

## Introduction

Parkinson’s disease (PD) is a chronic progressive neurodegenerative disease affecting over 1% of the population in their 60s (Wang et al., 2018). Its pathogenesis is still unclear, but genetics and environmental factors may play a major role (Koros et al., 2017; Bhat et al., 2018). Besides the characteristic motor symptoms, patients with PD also present a number of non-motor symptoms including sleep disorders, cognitive impairment, sensory dysfunction, and so on (Kalia and Lang, 2015). At present, the clinical diagnosis of PD mainly relies on some subjective evaluations, such as clinical symptoms, family history, and dopamine therapy response, leading to a misdiagnosis rate of 25% approximately (Mandal and Sairam, 2013). Therefore, it is very important to find an objective and efficient way to improve the diagnosis rate of PD.

Recently, high-resolution structural magnetic resonance imaging (MRI) has been frequently used to detect the subtle changes of the human brain. In patients with PD, numerous studies have found widespread brain atrophy in the cerebellar, subcortical, and cortical regions by using various structural MRI features, such as gray matter (GM) volume (GMV), white matter (WM) volume (WMV), and cortical thickness (CT). In addition, some cortical morphological features, including local gyrification index (LGI), local fractal dimension (LFD), and sulcal depth (SD), are also increasingly used to detect the structural changes of PD (Chaudhary et al., 2020; Li et al., 2020; Wang et al., 2021b). Compared with GMV, WMV, and CT, cortical morphological features can not only provide information about the shape of the cortical surface but also have higher accuracy and sensitivity in aging people, which may provide a new perspective to explore the neuropathological mechanism of PD (Li et al., 2020; Wang et al., 2021a). Furthermore, PD has a typical clinical phenomenon of lateral onset, which may be related to the asymmetry of the cortical or subcortical structures (Kim et al., 2014; Lee et al., 2015; Zhang et al., 2021). The asymmetry index (AI) can quantify the degree of structural asymmetry between the two hemispheres, providing a potential feature to characterize the brain of PD. However, based on the aforementioned structural MRI features, most of the previous studies have focused on exploring the structural differences between PD and normal controls (NCs) at the group level, which cannot assist in individualized diagnosis.

In recent years, machine learning (ML) technology based on structural MRI features has developed rapidly and showed huge advantages in assisting individualized diagnosis in various neurological and psychiatric diseases, such as Alzheimer’s disease (Wen et al., 2021), schizophrenia (Chand et al., 2020), and depression (Kang and Cho, 2020). Compared with traditional group-level analysis, ML technology takes inter-regional correlations into account, thereby providing increased sensitivity to subtle changes and spatially distribution differences (Zhu et al., 2019). In patients with PD, some structural features have been used to distinguish patients with PD and NCs. For example, by using the GMV, WMV, and cerebrospinal fluid (CSF) volume of substantia nigra, thalamus, hippocampus, frontal lobe, and midbrain, Rana et al. (2015b) found that the ML model based on the substantia nigra gained the highest accuracy, sensitivity, and specificity in differentiating patients with PD and NCs. In a multimodal ML study, Park et al. (2020) found patients with PD have GMV loss in basal ganglia, thalamus, cingulate cortex, insula, superior temporal cortex, and cerebellum. Furthermore, Morales et al. (2013) discovered that the CT of the entorhinal cortex was the most predictive region of the whole brain in the diagnosis of patients with PD with dementia. As far as we know, the LGI, LFD, SD, and AI features have not been used in the individualized diagnosis of patients with PD, and external validations were rarely observed in such studies to verify the validity of the model.

Hence, in this study, by using multiple structural MRI features, we constructed the cerebellar, subcortical, cortical, and the combined ML models to investigate their diagnostic efficacy and clinical net benefits, and the abnormal brain regions related to PD. Furthermore, in order to verify the validity of the model, external validation was employed in the study. We speculate that the combined ML model based on all features may gain the best classification performance compared to other models.

## Materials and Methods

### Subjects

Sixty patients with PD were recruited from the Neurology Department of the Second Affiliated Hospital of Soochow University and diagnosed based on the United Kingdom PD Brain Bank Criteria by an experienced neurologist. The exclusion criteria included (1) Parkinsonism-plus syndrome, such as multiple system atrophy and progressive supranuclear palsy; (2) Parkinson’s syndrome secondary to drugs, metabolic diseases, central nervous system infections, and head trauma; (3) significant leukoaraiosis performance; (4) dementia, brain tumors, stroke, and drug abuse; and (5) severe heart, liver, lung, and kidney diseases. In total, 56 age- and gender-matched NCs were also recruited, and they had no obvious cognitive impairment and neurological or mental illness. All subjects were right-handed and had no contraindications to MRI examination. The Unified Parkinson’s Disease Rating Scale-motor section (UPDRS-III) and Hoehn–Yahr stage (H–Y stage) were used to assess the severity of PD motor symptoms.

A total of 69 de novo patients with PD and 71 NCs from the Parkinson’s Progression Markers Initiative (PPMI)^1^ (Marek et al., 2011) dataset were also enrolled as an independent dataset for the external validation of the predictive models. PPMI is a public repository from various centers that provides neuroimaging and associated clinical information of various modes of patients with PD and matched NCs for identifying potential biomarkers of disease progression.

All participants in the study have received approval from the Institutional Review Board and have written the informed consent.

### MRI Data Acquisition

A Philips Achieva 3.0T MRI scanner (Philips, Best, Netherlands) with a 32-channel phased-array head coil was used to collect the 3D T1-weighted images. All subjects were in a supine position with their heads still and eyes closed. Memory pads and earplugs were used to reduce motion artifacts and scanning noise, respectively. The MRI scan parameters include: sagittal scan, repetition time (TR) = 7.1 ms, echo time (TE) = 3.5 ms, acquisition matrix size = 220 × 199, reconstruction matrix size = 352 × 352, flip angle = 8°, the field of view (FOV) = 220 mm^2^, slice thickness = 1.0 mm, slice gap = 0 mm, and slice number = 155.

### Extraction of Structural Features

First, MRIcron software^2^ was used to convert the MRI images from the DICOM format to the 3D NIfTI format. The images from all subjects were inspected by an experienced neuroradiologist, and no subjects were excluded from the analysis.

Then, the Ceres and volBrain (IBIME, Valencia, Spain^3^) online software were used to extract the cerebellar and subcortical features, respectively. Compared to similar software, Ceres and volBrain are more competitive in calculation time and accuracy (Manjón and Coupé, 2016; Romero et al., 2017). The processing steps included denoising, coarse inhomogeneity correction, Montreal Neurological Institute (MNI) affine registration, fine inhomogeneity correction, intensity normalization, and region of interest segmentation. Finally, the following features were obtained: the absolute and relative value of the whole volume, GMV and CT of the cerebellum (including the bilateral Lobule I-II, III, IV, V, VI, Crus I, Crus II, VIIB, VIIIA, VIIIB, IX, and X), and the subcortical volume (including the bilateral caudate nucleus, putamen, thalamus, globus pallidus, hippocampus, amygdala, nucleus accumbens, and the lateral ventricles). In addition, the AI was also obtained to evaluate the asymmetry degree of the aforementioned structures in the left and right hemispheres. The lower the absolute value, the smaller the asymmetry degree; when the two hemispheres are symmetrical, it is 0.

**FIGURE 1:**
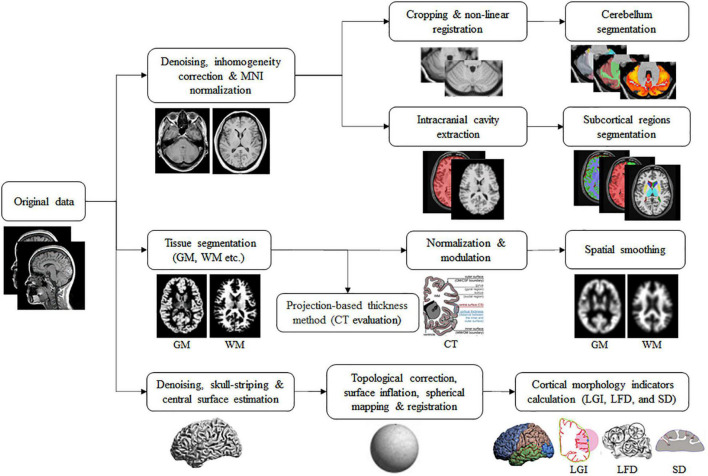
The flowchart of data processing and extraction steps.

The computational anatomy toolbox (CAT12,^4^ version r1733) in the statistical parametric mapping (SPM12,^5^ version 7771) was adopted to extract the cortical features. The extraction steps of GMV and WMV indicators were image segmentation, normalization and modulation (DARTEL algorithm), and space smoothing (8-mm Gaussian smoothing kernel). In addition, CAT12 used a projection-based thickness method to evaluate CT and the central surface in one step (Dahnke et al., 2013). Then, the central surface of the cortex was further used to calculate the LGI, LFD, and SD features based on the methods described in Luders et al. (2006), Yotter et al. (2011), and Yun et al. (2013), respectively. LGI can quantitatively assess the degree of gyrification at the vertex level, which represents the ratio of the area of the cortex located within folded regions to the total surface area. LFD can provide a quantitative description of structural complexity in the cerebral cortex, which could be a combination of sulcal depth, the frequency of cortical folding, and the convolution of gyral shape. SD refers to the linear distance from the inner surface of the brain (pia mater) to the outer surface based on the Euclidean distance. In this study, the automated anatomical labeling (AAL) template (80 regions, except for the cerebellar and subcortical regions) was used to extract the cortical volume of GM and WM and the Desikan-Killiany (DK40) (68 brain regions), a2009s (150 brain regions) and human connectome project multimodal parcellation (HCP-MMP) templates (360 brain regions) were used to extract the cortical CT, LGI, LFD, and SD features, respectively. The extraction process of all features is shown in Figure 1.

### Machine Learning Model Construction

#### Feature Selection

Pearson’s correlation test was used to remove redundant features between the two groups preliminarily. The significance threshold was set at 0.85. If there was a pair of features with | r| > 0.85, the one with a larger p-value calculated by the Mann–Whitney U test between the two groups was excluded. Then, the least absolute shrinkage and selection operator (LASSO) regression was used to further remove the irrelevant and redundant features (data dimensionality reduction), and the optimal penalty parameter tuning was conducted by 10-fold cross-validation, which was implemented in the Python programming environment using the scikit-learn package (Pedregosa et al., 2012). LASSO is a regularization technique, which penalizes the coefficients of the regression variables (add an L1-norm penalty term) and shrinks some of them to 0, thereby removing uninformative covariates and retaining the most predictive features (Lombardi et al., 2020). Compared with ridge regression (i.e., L2-norm), the LASSO regression has sparse feature selection characteristics, stronger robustness to outliers and noise changes, and higher calculation accuracy (Moreno et al., 2011). After Pearson’s correlation test, 88 cerebellar, 31 subcortical, and 637 cortical features were kept. The LASSO regression was applied to further reduce the dimensions of the features, and 5 cerebellar, 3 subcortical, and 4 cortical features with non-zero coefficients were finally selected for the model development (Supplementary Figure 1).

#### Model Construction

In this study, the cerebellar model, subcortical model, and cortical model based on corresponding features, separately, and the combined model integrating all selected features, were developed. All models were constructed and evaluated by using the logistic regression (LR) with the 5-fold cross-validation scheme in the development dataset, and their performances were also evaluated in the independent test dataset.

The LR classifier is a statistical modeling technique that estimates the probability of a dependent variable relating to a set of independent variables through the sigmoid function, and it has been widely used in biostatistics, medicine, econometrics, and other fields (Hsu et al., 2019).

The 5-fold cross-validation method was performed to validate the overall performance of the predictive models, in which the whole dataset was randomly partitioned into five subsets of similar size. Among these subsets, one subset was retained as the validation data for validating, and the remaining four subsets were used as the training data. The cross-validation procedure was repeated five times (each subset was used as validating data at least one time), and the averaged results were used to generate a single estimation.

### Diagnostic Performance and Clinical Utility Evaluation

The classification results were assessed in terms of sensitivity (Se), specificity (Sp), positive predictive value (PPV), and negative predictive value (NPV). Their calculation formulas are defined as follows:


Se=TP/(TP+FN)

Sp=TN/(TN+FP)

PPV=TP/(TP+FP)

NPV=TN/(TN+FN)

where true positive (TP) and true negative (TN) are the number of cases that correctly divided into PD and NC groups, respectively; false positive (FP) was the number of NCs incorrectly identified as having PD; false negative (FN) was the number of patients with PD incorrectly identified as NCs. Se and Sp represent the proportion of cases correctly classified into PD and NC groups, respectively. PPV represents the probability that the ML classification result is positive for PD, whereas NPV represents that is negative for NCs.

In the classification, the receiver operating characteristic (ROC) curve was performed to determine the performance of the classifiers by plotting the rate of sensitivity and 1 − Sp. The area under the curve (AUC) was used to evaluate the classification performance of the models. The larger the AUC value (range 0–1), the better the model classification performance.

The consistency between the predicted PD probability and actual PD rate was evaluated through calibration curve by using 1,000 times bootstrapping resampling method, and the Hosmer–Lemeshow test was conducted to assess the goodness-of-fit of the predictive models in both training and validation datasets (Finazzi et al., 2011).

**FIGURE 2:**
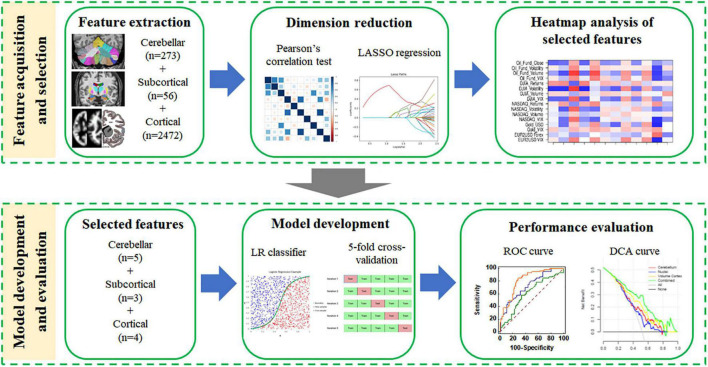
The flowchart of the machine learning steps. Feature selection (data dimensionality reduction), model building (using the optimal discrimination features set), and model evaluation were performed using the extracted structural features.

Decision curve analysis (DCA) is a graphical statistical method, which can evaluate the clinical net benefit of the selected model at different threshold probabilities in the validation set (Liu et al., 2017). Unlike traditional performance evaluation methods (such as the ROC curve), it considers the clinical utility of specific models (two or more) and integrates the preferences of patients or decision-makers into the analysis (Fitzgerald et al., 2015). The DCA method can identify risk models for better clinical decision-making, and it is worthy of further promotion and application in clinical analysis. The illustration for the study design and the development of machined learning-based predictive models is presented in Figure 2.

### Statistical Analysis

The demographic and clinical data were statistically analyzed by SPSS 25.0 (IBM Corporation, Armonk, NY, United States). After the normality test, the data conforming to the normal distribution (expressed as mean ± standard deviation, x̄ ± s) was tested for homogeneity of variance. Demographic and clinical data were analyzed with the independent-sample t-test for continuous variables and the chi-square test for categorical variables. The significance level was set at p < 0.05. As mentioned in Subsection “Diagnostic Performance and Clinical Utility Evaluation”, the ROC curve was plotted, and Se, Sp, PPV, NPV, and AUC values were calculated to evaluate the diagnostic efficiency of each ML model. The difference between two AUC values of different models in the same dataset was compared to Delong’s test (DeLong et al., 1988). The Hanley and McNeil test was used for the comparison of two AUC values from different datasets (Hanley and McNeil, 1983). The HemI software (version 1.0) was used to plot the heatmap of selected features. The calibration curve was plotted using the “rms” package, and the decision curve was plotted using the “rmda” package.

## Results

### Demographic and Clinical Data

No significant difference was observed with respect to the age (p = 0.211 and p = 0.276), gender (p = 0.564 and p = 0.958), and education (p = 0.351 and p = 0.455) between patients with PD and NCs in the development and independent test datasets, respectively, as shown in Table 1.

**TABLE 1:** Demographic and clinical data of subjects in the development and independent test datasets.

Variables	Development dataset			Independent test dataset		
	PD (*n* = 60)	NC (*n* = 56)	*p*	PD (*n* = 69)	NC (*n* = 71)	*p*
Age (years)	61.60 ± 6.93	63.07 ± 5.54	0.211	61.01 ± 9.89	59.10 ± 10.82	0.276
Gender (M/F)	(30/30)	(31/25)	0.564	(45/24)	(46/25)	0.958
Education (years)	7.88 ± 3.76	8.55 ± 3.94	0.351	15.30 ± 2.84	15.66 ± 2.81	0.455
Duration of illness (years)	3.73 ± 2.05	–	–	0.57 ± 0.53	–	–
UPDRS III score	22.55 ± 12.52	–	–	20.46 ± 9.21	–	–
H-Y stage	1.91 ± 0.63	–	–	1.52 ± 0.50	–	–

*PD, Parkinson’s disease; NC, Normal control; UPDRS-III, Unified Parkinson’s Disease Rating Scale-motor section; H–Y stage, Hoehn and Yahr stage.*

### Feature Selection Results

**FIGURE 3:**
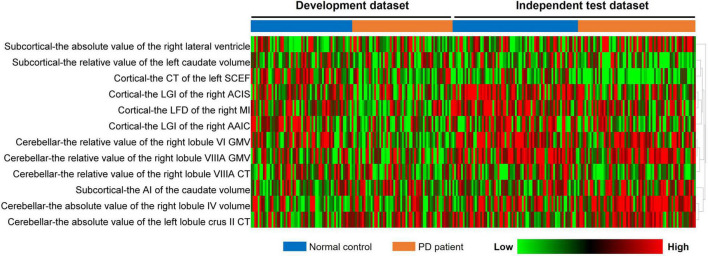
Heatmap analysis of the selected features in both datasets. Each column corresponds to one label (PD or NCs), and each row represented a structural feature; red represents a relatively high feature value, and the green represents a relatively low feature value. PD, Parkinson’s disease; NC, normal control.

A total of 273 cerebellar, 56 subcortical, and 2,472 cortical features were originally extracted from the structural MRI of both groups. After the two-step feature selection of Pearson’s correlation test and LASSO regression, 5 cerebellar, 3 subcortical, and 4 cortical features were finally retained as the most discriminative regions between PD and NC groups. The analysis of these selected features in the development and independent test datasets is summarized in Table 2. These regions included the cerebellar (the absolute value of the left lobule crus II CT, the relative value of the right lobule VIIIA CT, the relative value of the right lobule VI/VIIIA GMV, and the absolute value of the right lobule IV volume), the subcortical (the AI of the caudate volume, the relative value of the left caudate volume, and the absolute value of the right lateral ventricle), and the cortical features [the LGI of the right anterior circular insular sulcus (ACIS) and anterior agranular insula complex (AAIC), the LFD of the right middle insular (MI) area, and the CT of the left supplementary and cingulate eye field (SCEF)]. In addition, the heatmap of these selected features was drawn based on the normalized feature value (Figure 3).

**TABLE 2:** Comparison of the selected features in the development and independent test datasets.

Model	Key features	Development dataset			Independent test dataset		
		NC ($\bar{\text{x}}\pm \text{s}$)	PD ($\bar{\text{x}}\pm \text{s}$)	*p*	NC ($\bar{\text{x}}\pm \text{s}$)	PD ($\bar{\text{x}}\pm \text{s}$)	*p*
Cerebellar	The absolute value of the left lobule crus II CT	–0.088 ± 0.922	0.082 ± 1.061	0.362	0.066 ± 0.987	0.487 ± 0.766	0.006
	The relative value of the right lobule VIIIA CT	0.089 ± 1.088	–0.083 ± 0.902	0.358	0.105 ± 0.891	–0.286 ± 0.860	0.010
	The relative value of the right lobule VIIIA GMV	0.097 ± 0.952	–0.091 ± 1.034	0.317	1.021 ± 1.393	0.565 ± 0.902	0.024
	The relative value of the right lobule VI GMV	0.268 ± 0.973	–0.251 ± 0.959	0.005	0.807 ± 1.134	0.647 ± 1.157	0.415
	The absolute value of the right lobule IV volume	–0.069 ± 1.036	0.064 ± 0.961	0.480	0.336 ± 1.061	0.891 ± 0.957	0.002
Subcortical	The AI of the caudate volume	–0.044 ± 0.900	0.041 ± 1.083	0.654	–0.031 ± 1.041	0.508 ± 2.028	0.051
	The relative value of the left caudate volume	0.101 ± 0.944	–0.094 ± 1.041	0.299	–0.070 ± 0.985	–0.476 ± 1.100	0.024
	The absolute value of the right lateral ventricle	–0.092 ± 0.939	0.086 ± 1.047	0.342	0.302 ± 1.702	0.882 ± 2.087	0.075
Cortical	The LGI of the right ACIS	0.219 ± 0.796	–0.204 ± 1.121	0.023	0.750 ± 0.966	0.224 ± 1.129	0.004
	The LGI of the right AAIC	0.307 ± 0.935	–0.286 ± 0.974	0.001	0.265 ± 1.129	–0.103 ± 1.097	0.054
	The LFD of the right MI	0.299 ± 0.869	–0.279 ± 1.033	0.002	0.688 ± 1.036	0.240 ± 1.061	0.013
	The CT of the left SCEF	0.316 ± 0.988	–0.295 ± 0.917	0.001	-0.315 ± 0.959	–0.775 ± 0.791	0.003

($\bar{x}\pm s$): *normalized mean ± standard deviation, value range [−1, 1]. CT, cortical thickness; GMV, gray matter volume; AI, asymmetry index; LGI, local gyrification index; ACIS, anterior circular insular sulcus; AAIC, anterior agranular insula complex; LFD, local fractal dimension; MI, middle insular area; SCEF, supplementary and cingulate eye field.*

### Model Evaluation Results

**FIGURE 4:**
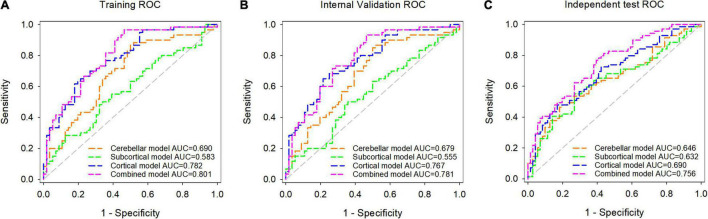
Receiver operating characteristic (ROC) analysis of the cerebellar model, subcortical model, cortical model, and combined model in the training dataset **(A)**, internal validation dataset **(B)**, and independent test dataset **(C)**.

The ROC curves and Se, Sp, PPV, NPV, and AUC values of each model in the training, internal validation, and independent test datasets are shown in Figure 4 and Table 3. The AUC values of the cerebellar model, subcortical model, and cortical model were 0.679, 0.555, and 0.767 in the internal validation dataset, respectively. The combined model had achieved an AUC value of 0.781 in the internal validation dataset, which was higher than that of the cerebellar (p-value = 0.027), subcortical (p-value < 0.001), and cortical model (p-value = 0.473). Similar results were observed in the independent test dataset, with the corresponding AUC values of the cerebellar, subcortical, cortical, and combined models achieving 0.646, 0.632, 0.690, and 0.756, respectively. The combined model also outperformed the other three models (Delong’s test, all p-values < 0.05). These results suggested that the combined model had the highest diagnostic efficiency, followed by the cortical model. The combined model also showed good robustness, as the Hanley and McNeil test suggested no significant difference between the AUC values on the internal and external datasets (p = 0.672).

**TABLE 3:** Detailed performance of the predictive models in the training, internal validation, and independent test datasets.

Dataset	Model	AUC (95% CI)	*P*	Threshold	Se (%)	Sp (%)	PPV (%)	NPV (%)
Training	Cerebellar	0.690 (0.598–0.773)	0.017	>0.4535	86.67	50.00	65.00	77.78
	Subcortical	0.583 (0.488–0.674)	<0.001	>0.5280	48.33	67.86	61.70	55.07
	Cortical	0.782 (0.696–0.853)	0.352	>0.6092	61.67	82.14	78.72	66.67
	Combined	0.801 (0.717–0.870)	reference	>0.3496	96.67	53.57	69.05	93.75
Internal validation	Cerebellar	0.679 (0.586–0.763)	0.027	>0.4642	85.00	50.00	64.56	75.68
	Subcortical	0.555 (0.460–0.647)	<0.001	>0.5271	48.33	66.07	60.42	54.41
	Cortical	0.767 (0.679–0.840)	0.473	>0.5896	65.00	78.57	76.47	67.69
	Combined	0.781 (0.694–0.852)	reference	>0.3823	93.33	53.57	68.29	88.24
Independent test	Cerebellar	0.646 (0.560–0.725)	0.043	>0.5314	47.83	80.28	70.21	61.29
	Subcortical	0.632 (0.547–0.712)	0.024	>0.5332	55.07	73.24	66.67	62.65
	Cortical	0.690 (0.606–0.765)	0.008	>0.5959	47.83	83.10	73.33	62.11
	Combined	0.756 (0.677–0.825)	reference	>0.4413	82.61	60.56	67.06	78.18

*Se, sensitivity; Sp, specificity; PPV, positive predict value; NPV, negative predict value; reference, comparison reference for the Delong’s test.*

The non-significant statistic of the Hosmer–Lemeshow test suggested that all models showed no significant deviation from an ideal fitting (p-values = 0.552, 0.917, 0.358, and 0.618 for the cerebellar, subcortical, cortical, and combined models in the internal validation dataset, and p-values = 0.423, 0.648, 0.579, and 0.212 for the cerebellar, subcortical, cortical, and combined models in the independent test dataset, respectively). The calibration curves of these models in the internal validation and independent test datasets are shown in Supplementary Figures 2, 3, respectively.

**FIGURE 5:**
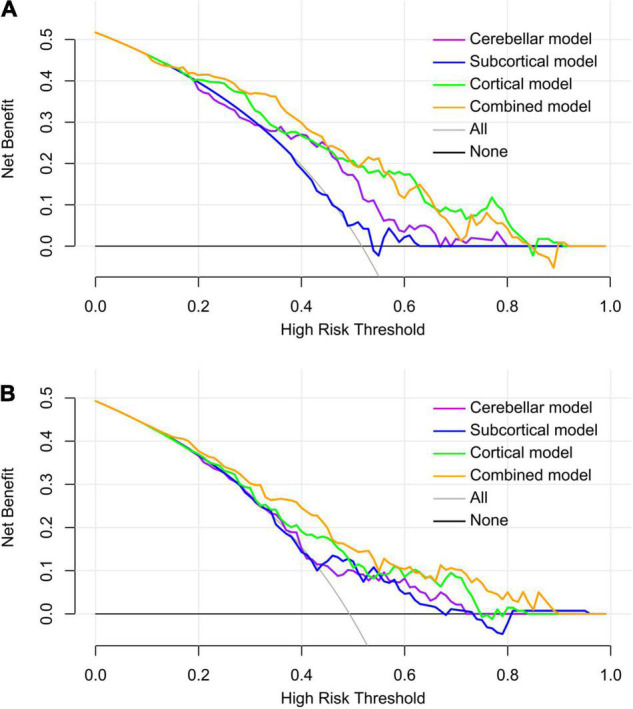
Decision curve analysis of the predictive models in the internal validation dataset **(A)** and the independent test dataset **(B)**. Within a larger threshold probability range, the combined model has the highest clinical net benefit, followed by the cortical model.

The DCA curves of each model based on both classifiers are shown in Figure 5. Quantitative analysis of the clinical net benefit under different threshold probabilities in the internal validation dataset and the independent test dataset demonstrated that the combined model using all the selected features showed higher net benefit than that of other models (internal validation dataset, 15–50%; independent test dataset, 10–60% and 65–90%), followed by the cortical model.

## Discussion

Using multiple structural MRI features of the whole brain and ML technology, the present study revealed that the combined model based on all features obtained the highest diagnostic efficiency and clinical application value. The most discriminative regions between PD and NC groups included the cerebellar (the absolute value of the left lobule crus II CT, the relative value of the right lobule VIIIA CT, the relative value of the right lobule VI/VIIIA GMV, and the absolute value of the right lobule IV volume), the subcortical (the AI of the caudate volume, the relative value of the left caudate volume, and the absolute value of the right lateral ventricle), and the cortical features (the LGI of the right ACIS and AAIC, the LFD of the right MI, and the CT of the left SCEF).

Compared to other single models, the combined model significantly improved the diagnostic efficiency and clinical net benefit in the development and independent test datasets, which may highlight the importance of multiple structural features in ML research. Similar to our study, some previous studies also found the superiority of employing multiple features in the ML studies. For example, Rana et al. (2016) and Cigdem and Demirel (2018) revealed that modeling after fusing GM and WM features achieved higher accuracy than using a single feature, with an improvement of up to about 15 and 30% in accuracy, respectively. By using multiple structural features (GM, WM, CSF, CT, surface area, and the correlation index of CT and surface area), Peng et al. (2017) adopted filter- and wrapper-based feature selection method and the support vector machine classifier and obtained the highest accuracy of 85.78%. Hence, it is speculated that multiple structural features may provide more comprehensive information of the brain from different perspectives than the single feature. In our study, the cortical morphological features (LGI, LFD, and SD) were first introduced into the ML models in patients with PD. Changes in these cortical morphological features may result from the combined effects of GM, WM, and corticortical connections, likely reflecting more subtle changes of the brain (Van Essen, 1997; Im et al., 2008). In the cortical model, four cortical morphological features but no GM or WM features were retained as the most predictive features. Hence, our results suggested that cortical morphological features may have higher sensitivity than volume features in PD diagnosis. Moreover, external validation was adopted to verify the robustness and reproducibility of these models, which may not be employed in previous PD ML studies, and the results suggested that the combined model performed well and would be helpful in assisting clinical diagnosis of PD.

This study found that the most discriminating cerebellar features of patients with PD included the absolute value of the left lobule crus II CT, the relative value of the right lobule VIIIA CT, the relative value of the right lobule VI/VIIIA GMV, and the absolute value of the right lobule IV volume. The cerebellum not only plays a crucial role in motor function but also is responsible for cognition and emotion processes (Li et al., 2019; Xu et al., 2019). Many studies have suggested the abnormal structure and function of the cerebellum in patients with PD (Deng et al., 2016; Ma et al., 2018; Li et al., 2019; Xu et al., 2019; Park et al., 2020). Furthermore, in an ML study based on the volumetric features of the cerebellum, Zeng et al. (2017) gained excellent classification performance of accuracy > 95% between patients with PD and NCs, which highlighted the importance of the cerebellum in PD diagnosis. Compared to Zeng’s research, our classification results were not very satisfactory. The discrepancies among these results may be related to the heterogeneity of patients with PD and the variations in methodological approaches. For example, most of the patients with PD in our study had a shorter duration of disease relative to theirs(3.73 ± 2.05 years vs. 5.0 ± 2.4 years); hence, the changes in brain structure may not be obvious. More importantly, although satisfactory results were obtained, the stability of their model was unknown; in contrast, we additionally performed external validation to make the model more reliable.

As to the subcortical regions, the caudate nucleus was the most discriminating structure between both groups. As a part of the striatum, the caudate nucleus is primarily involved in emotion regulation, reward processing, decision-making, and executive functioning (Owens-Walton et al., 2018). Many previous ML studies have found its atrophy in patients with PD (Long et al., 2012; Rana et al., 2015a,^2016^; Adeli et al., 2016; Liu et al., 2018; Song et al., 2021). The asymmetry of brain structure is related to various aspects of language function, visual spatial tasks, attention, and emotion, and it changes with aging (Sarica et al., 2018). PD has a typical clinical phenomenon of lateral onset, and some studies have found that the brain structural changes of PD also have laterality (Kim et al., 2014; Claassen et al., 2016), which implies that the AI has potential application value in PD diagnosis. Additionally, the volume of the right lateral ventricle also differed between the two groups. In brief, the expansion of the lateral ventricle likely reflects atrophy of the basal ganglia (Kocaman et al., 2019), which has been reported in several previous ML and group-level researches in patients with PD (Rana et al., 2015a,^2016^; Solana-Lavalle and Rosas-Romero, 2021; Song et al., 2021). As an indirect sign, the enlargement of the lateral ventricle may provide vital reference information for the macroscopic structural changes of the PD brain.

In the cortical model, the LGI of the right ACIS and AAIC, the LFD of the right MI, and the CT of the left SCEF were retained as the most discriminating features. The insula (including ACIS, AAIC, and MI) is a hub region of the salience network and is mainly involved with a variety of brain functions including perception, emotion, and cognition (Huang et al., 2020). A number of ML studies have reported abnormalities of the insula in patients with PD (Babu et al., 2014; Rana et al., 2015a,^2016^; Adeli et al., 2016; Peng et al., 2017; Park et al., 2020). SCEF is a part of the supplementary motor area, which is primarily related to the production of autonomous, complex, and continuous movement (Jubault et al., 2011). Similar to our results, some studies have also discovered the CT thinning of the supplementary motor area in patients with PD (Jubault et al., 2011; Hanganu et al., 2014; Guimarães et al., 2016). Additionally, volume atrophy of the supplementary motor area was also detected by some ML research works (Adeli et al., 2016; Peng et al., 2017; Tang et al., 2018). Hence, these studies have shown that the insula and supplementary motor area play an important role in the pathogenesis of PD and could be used as the discriminating regions in PD diagnosis.

This study has some limitations. First, the sample size is relatively small, the expansion of samples is still needed to verify the reliability of these experimental models. Second, this study did not analyze the subtypes of patients with PD based on clinical symptoms, and we will expand the sample size for further research to understand the prognosis of various subtypes. Finally, although we have fully explored the multiple structural MRI features, ML studies based on multimodal MRI, such as diffusion and functional MRI, may further improve the classification performance.

## Conclusion

In conclusion, the construction of ML models from different perspectives based on multiple structural MRI indicators has high diagnostic efficiency and clinical net benefits for PD in both internal and external validation datasets, among which the combined model performed best, followed by the cortical model. The most diagnostic discriminating brain regions identified by ML are expected to be served as potential neuroanatomical markers of PD, further deepening our understanding of its pathogenesis. The combined ML model based on multiple indicators may be of great value in assisting the clinical diagnosis of PD and may become an effective and clinically applicable new method.

## Data Availability Statement

The raw data supporting the conclusions of this article will be made available by the authors, without undue reservation.

## Ethics Statement

The studies involving human participants were reviewed and approved by the Medical Research Ethical Committee of the Second Affiliated Hospital of Soochow University (Suzhou, China) and the Michael J. Fox Foundation for Parkinson’s Research. The patients/participants provided their written informed consent to participate in this study.

## Author Contributions

YY and LJ have contributed equally to this work and share the first authorship, analyzed the data, plotted tables, and wrote this article. EW and GF designed and organized the study. YJ, NZ, ZJ, HY, CM, and WL collected and analyzed the data. All authors contributed to manuscript revision, read, and approved the submitted version.

## Conflict of Interest

HY was employed by the Infervision Medical Technology Co., Ltd, Beijing, China. The remaining authors declare that the research was conducted in the absence of any commercial or financial relationships that could be construed as a potential conflict of interest.

## Publisher’s Note

All claims expressed in this article are solely those of the authors and do not necessarily represent those of their affiliated organizations, or those of the publisher, the editors and the reviewers. Any product that may be evaluated in this article, or claim that may be made by its manufacturer, is not guaranteed or endorsed by the publisher.

## Footnotes

1
https://www.ppmi-info.org/data

2
https://www.nitrc.org/projects/mricron

3
https://www.volbrain.upv.es/index.php

4
http://www.neuro.uni-jena.de/cat12/

5
http://www.fil.ion.ucl.ac.uk/spm/software/spm12/
