# Supplementary material for: Machine Learning Models for Diagnosis of Parkinson’s Disease Using Multiple Structural Magnetic Resonance Imaging Features
Source: Front Aging Neurosci. 2022 Apr 13;14:808520. doi: 10.3389/fnagi.2022.808520 (PMC9043762; doi:10.3389/fnagi.2022.808520)
Supplement: Supplementary file 1 [file Data_Sheet_1.PDF]

Supplementary figures

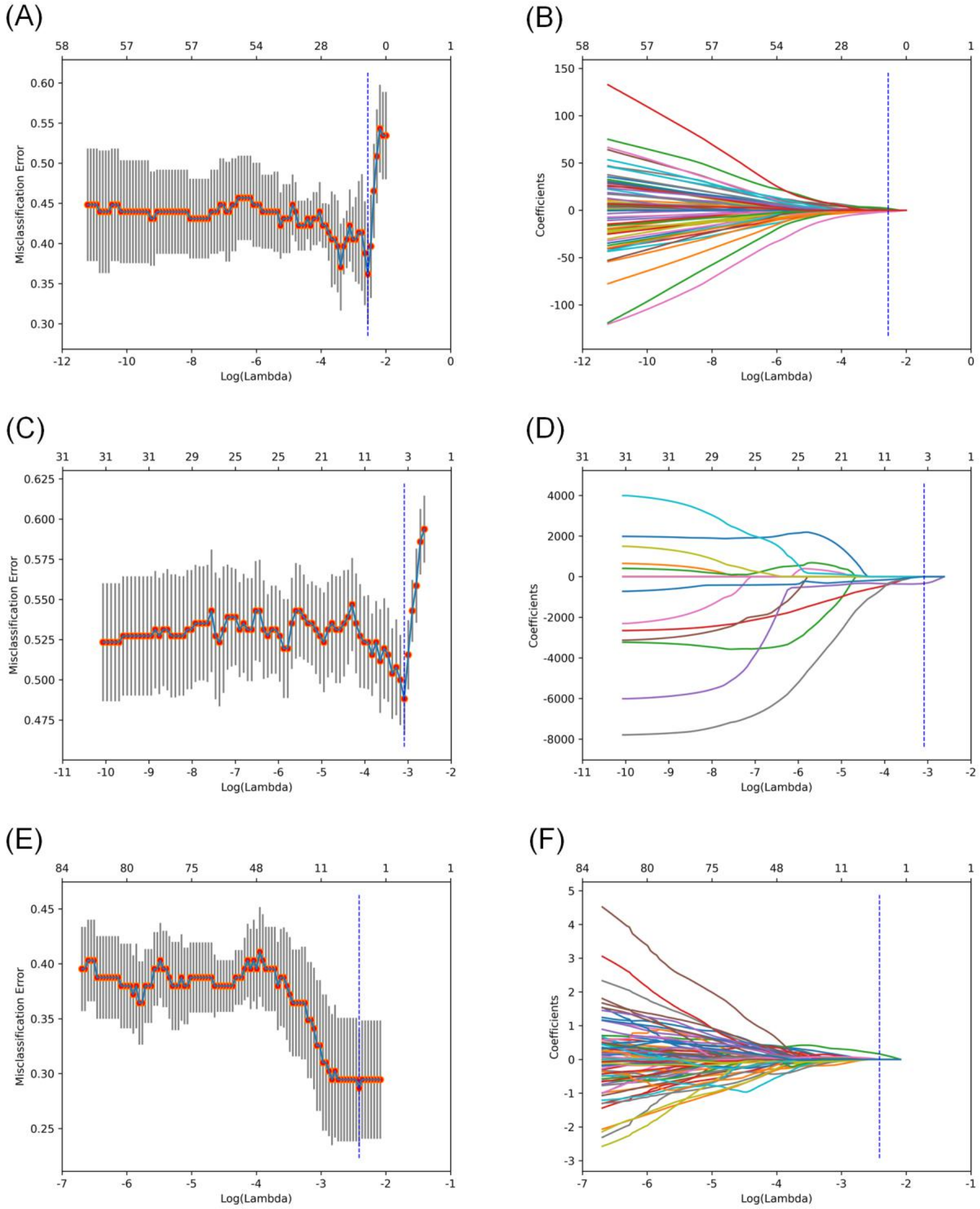

Supplementary Figure 1. LASSO regression-based feature selection. The variance threshold approach-based dimensionality reduction profile showed that the optimal log(lambda) values were -2.5576, -3.0871 and -2.4132 for the cerebellar features (A), subcortical features (C) and cortical features (E), respectively. There were 5, 3 and 4 non-zero coefficients against the optimal log(lambda) sequence for the cerebellar features (B), subcortical features (D) and cortical features (F), respectively.

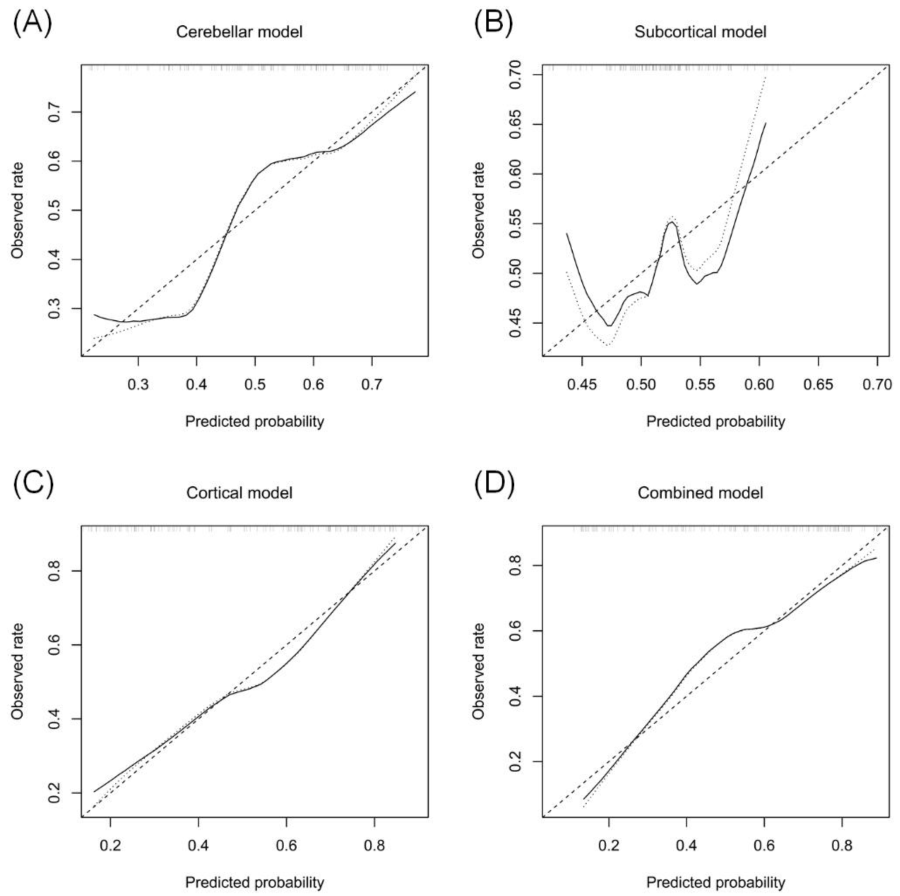

Supplementary Figure 2. Calibration curve analysis of the Cerebellar model (A), Subcortical model (B), Cortical model (C) and Combined model (D) in the internal validation dataset.

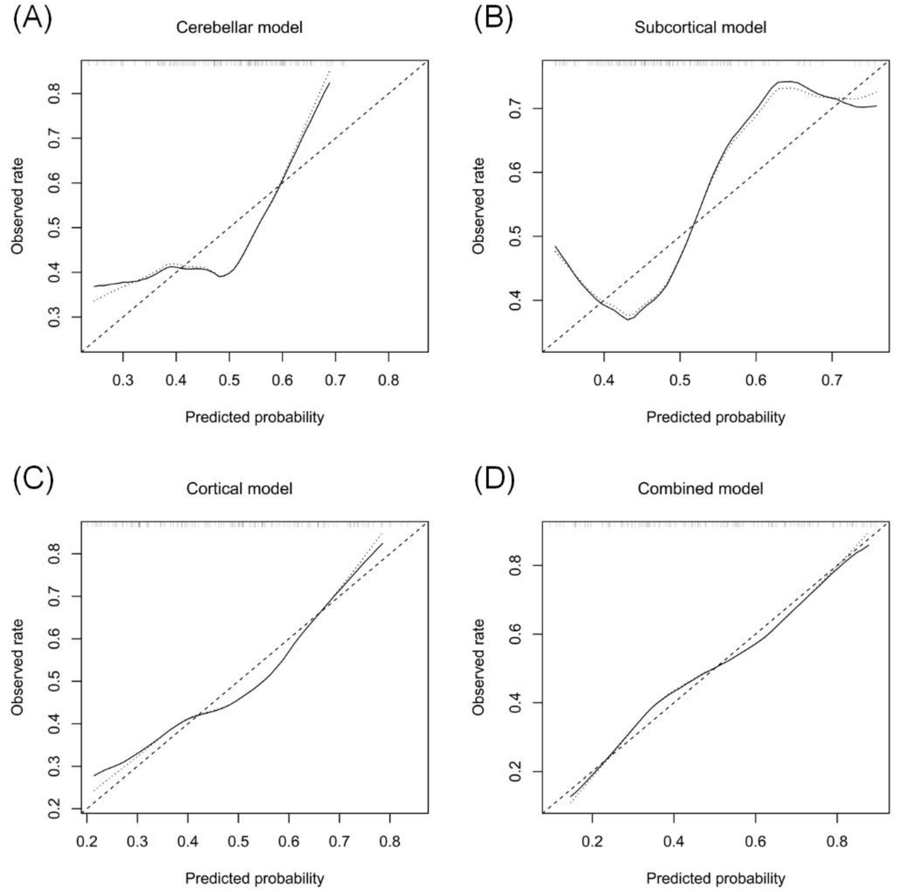

Supplementary Figure 3. Calibration curve analysis of the Cerebellar model (A), Subcortical model (B), Cortical model (C) and Combined model (D) in the independent test dataset.
